# A Bacteriophage Protein-Based Impedimetric Electrochemical Biosensor for the Detection of *Campylobacter jejuni*

**DOI:** 10.3390/bios14080402

**Published:** 2024-08-21

**Authors:** Baviththira Suganthan, Ashley M. Rogers, Clay S. Crippen, Hamid Asadi, Or Zolti, Christine M. Szymanski, Ramaraja P. Ramasamy

**Affiliations:** 1 Nano Electrochemistry Laboratory, School of Chemical, Materials and Biomedical Engineering, University of Georgia, Athens, GA 30602, USA; baviththira.suganthan@uga.edu (B.S.); or.zolti@uga.edu (O.Z.); 2 Department of Microbiology, University of Georgia, Athens, GA 30602, USA; ashley.rogers123@uga.edu (A.M.R.); cszymans@uga.edu (C.M.S.); 3 Complex Carbohydrate Research Center, University of Georgia, Athens, GA 30602, USA

**Keywords:** food safety, impedance, phage protein, foodborne pathogen

## Abstract

*Campylobacter jejuni* is a common foodborne pathogen found in poultry that can cause severe life-threatening illnesses in humans. It is important to detect this pathogen in food to manage foodborne outbreaks. This study reports a novel impedimetric phage protein-based biosensor to detect *C. jejuni* NCTC 11168 at 100 CFU/mL concentrations using a genetically engineered receptor-binding phage protein, FlaGrab, as a bioreceptor. The electrochemical impedance spectroscopy (EIS) technique was employed to measure changes in resistance upon interaction with *C. jejuni*. The sensitivity of the phage protein-immobilized electrode was assessed using the various concentrations of *C. jejuni* NCTC 11168 ranging from 10^2^–10^9^ colony forming units (CFU)/mL). The change transfer resistance of the biosensor increased with increasing numbers of *C. jejuni* NCTC 11168 cells. The detection limit was determined to be approximately 10^3^ CFU/mL in the buffer and 10^2^ CFU/mL in the ex vivo samples. *Salmonella enterica* subsp. *enterica* serotype Typhimurium-291RH and *Listeria monocytogenes* Scott A were used as nontarget bacterial cells to assess the specificity of the developed biosensor. Results showed that the developed biosensor was highly specific toward the target *C. jejuni* NCTC 11168, as no signal was observed for the nontarget bacterial cells.

## 1. Introduction

*Campylobacter jejuni*, a microaerophilic, gram-negative bacteria, is a common foodborne pathogen in poultry that can cause severe life-threatening illnesses in humans [1]. The infectious dose of *C. jejuni* is low, and less than 500 CFU/mL is enough to cause the disease [2,3]. Besides causing bloody diarrhea, being infected by this pathogen can also result in Guillain–Barré syndrome (GBS), which is an autoimmune disorder that damages the peripheral nervous system. The flagella of *C. jejuni* play a crucial role in colonizing the human gastrointestinal tract. The flagellar filaments are composed of approximately 20,000 flagellin subunits [4]. The flagellar filament is driven by the proton motive force (PMF) generated by a multi-subunit motor protein [5]. Proper flagella formation and normal filament movement in *Campylobacter* require a glycosylated flagella [6,7]. Researchers from the Economic Research Service estimated that foodborne pathogens cost the U.S. economy USD 15.5 billion annually, and *Campylobacter* infections cost USD 1.9 billion [8]. This estimate only includes the cost associated with medical care, time lost from work, and losses due to death. The inclusion of costs associated with food product recalls, loss of consumer interest, and other unaccounted costs, could drive the economic losses due to foodborne pathogens much higher. Therefore, it is important to develop accurate and robust detection methods to isolate and enumerate pathogens in the food source as early as possible in the food supply chain. Even though predominant conventional detection methods, such as standard microbiological and biochemical tests, are highly reliable, inexpensive, sensitive, and allow both the qualitative and quantitative assessment of pathogens, these methods require laboratory space and skilled labor and are time consuming. Current detection methods use a nonselective medium to revive the cells from the food matrix, and then a selective medium is used to identify the target pathogen. Finally, biochemical tests are used to confirm the species’ identity further. This process typically requires 48 h to several days, depending on the nature of the food sample and the type of target microorganisms. This longer testing time in traditional methods contributes to a significant time lag between the first outbreak of foodborne pathogens to its final identification and verification [9,10]. This is considered a major drawback in terms of the rapid identification of microorganisms in contaminated food samples [11,12].

Electrochemical biosensors offer distinct advantages for detecting foodborne pathogens. Their low cost, sensitivity, simplicity, and ability to be easily miniaturized, make them particularly suitable for this purpose. They are also portable, enabling onsite tracking and boasting fast detection times. Moreover, they do not require specific expertise, require minimal sample dosage, and allow for multicomponent measurement. In addition, they provide real-time and quantitative pathogen detection, unlike other methods that take longer and only provide qualitative data [13,14,15,16,17,18,19,20,21].

The specificity of the biosensor is considered one of the most important criteria, and it is highly dependent on the type of biorecognition element used. Even though antibodies and enzymes pose high selectivity, they are susceptible to pH and temperature variations.

Biosensors based on phages and receptor-binding phage proteins (RBPPs) are emerging as a viable alternative for nucleic acid and antibody-based biosensors. Bacteriophage-based biosensors are accurate and specific due to their high specificity towards the target organisms [22,23,24,25]. However, using the whole bacteriophage has a higher chance of triggering resistance to phage infection as compared to the use of RBPPs. Studies show that RBPPs show better resistance against proteases and better stability against environmental factors such as pH and temperature [26]. RBPPs are smaller in size than phages. This would allow more RBPPs per unit surface area than phage particles. This would allow more target bacteria binding per unit surface area and thereby increase the sensitivity of the test. The possibility of having a large number of RBPPs per unit surface area would also allow the detection of multiple pathogens simultaneously using specific RBPPs without compromising sensitivity. In addition, naturally occurring phages would proceed with the infection cycle and lyse the target bacterial cells. Newly released phages from the lysed bacterial cell would also compete with phages immobilized on the electrode. This would negatively affect the accuracy and sensitivity of the detection. Further, RBPPs could be modified with tags without altering their binding affinity, and those tags could be used not only for purification but also to orient RBPPs on the biosensor electrode surface. These advantages offered by RBPPs make them an attractive alternative to phage-based biosensors.

FlaGrab (formerly GST-CCGp047) is an RBPP expressed by the NCTC 12673 phage [1]. FlaGrab binds to the acetamidino-modified pseudaminic acid (Pse5Ac7Am) glycan displayed on the flagellin subunits of *C. jejuni* flagella. Even though the amino acid sequence of the protein and its structural features are known, the conserved domains or critical amino acids involved in receptor binding have not been reported yet. This protein recognizes flagellin from several strains of *C. jejuni* and *Campylobacter coli* [27], the two *Campylobacter* species most commonly associated with human infection. Proteomic analyses and antibody recognition tests do not show the presence of FlaGrab on the phage virion, indicating a nonstructural role of the protein [28,29,30], possibly in the reduction of bacterial motility. The binding domain of FlaGrab is located in the C-terminal quarter of the protein (CC-FlaGrab). Therefore, the bioreceptor’s size could be further reduced to increase the number of molecules per unit area and sensitivity of the biosensor. Even though CC-FlaGrab harbors bacteriostatic properties, it does not lyse the bacterial cells, making it a better alternative to phages. The availability of a genetically engineered CC-FlaGrab with a GST tag would allow better purification and better orientation of the protein between the biosensor and bacteria receptor. Immobilization of the protein could be targeted towards the GST tag, thereby minimizing the obstruction of critical amino acids involved in the interaction with the receptor. It is known that the GST-tagged FlaGrab is compatible with immobilization on other surfaces, and immobilization could be performed without affecting the interaction with the target organism. These properties make FlaGrab a suitable bioreceptor for biosensors [31].

This paper describes developing an impedimetric biosensor architecture to detect *C. jejuni* using RBPP as the biorecognition molecule. The biosensor architecture comprises multiwalled, nanostructured, carbon nanotube-based glassy carbon electrodes. 1-pyrenebutanoic acid, succinimidyl ester (PBSE) was used as a molecular linker to immobilize the RBPP on the electrode. The response of the biosensor was measured based on the monitoring of the change in the charge-transfer resistance, resulting in the binding of *C. jejuni* to the RBPP. Further, *L. monocytogenes* Scott A and ser. Typhimurium-291RH were used as nontarget pathogens to evaluate the specificity of the biosensor.

Over the past 25 years, only a few studies have reported using electrochemical biosensors to detect *C. jejuni* (Table 1). These studies have mainly used antibodies as bio-receptors for detecting *C. jejuni*. While a surface plasma resonance (SPR) sensor system has been employed for the *Campylobacter* phage protein-based detection of *C. jejuni*, its reliance on sophisticated SPR equipment makes this approach prohibitively expensive [31]. In contrast, our impedimetric-based biosensor architecture for detecting *C. jejuni* offers a user friendly and cost-effective alternative to SPR-based methods.

## 2. Materials and Methods

### 2.1. Materials

Multiwalled carbon nanotubes (MWCNTs) with an outer diameter of 20–30 nm and a length of 10–30 µm were purchased from Cheap Tubes Inc. Silica polishing powder (0.05-micron type N gamma silica powder) was purchased from Electron Microscopy Sciences. Working electrode [glassy carbon electrode (part number CHI104)], reference electrode [Ag/AgCl (part number CHI111)], and counter electrode [platinum wire (part number CHI115)] were purchased from CH Instruments, Inc., Austin, TX, USA. BL21(DE3) Competent *E. coli* cells were purchased from New England Biolabs. The Glutathione-S-Transferase column (GST column) for protein purification was purchased from GE Healthcare (GSTrapTM HP).

Mueller–Hinton agar (MH) and brain heart infusion (BHI) were sourced from Hardy Diagnostics CRITERION^TM^. 1-pyrenebutanoic acid succinimidyl ester (PBSE), bovine serum albumin (BSA), and dimethylformamide (DMF) were obtained from Sigma-Aldrich. Disodium phosphate (Na_2_HPO_4_) was procured from Research Products International Corp, while sodium chloride (NaCl) was from EMD chemicals. Magnesium sulfate heptahydrate (MgSO_4_·7H_2_O) was acquired from J.T. Baker, and potassium phosphate dibasic (KH_2_PO_4_) and potassium chloride were from BDH. Tris base, tryptone, ethanol, glutathione, and protease inhibitor cocktail (100x) were purchased from Fisher Scientific. Yeast extract and agar powder were sourced from Becton Dickinson and Company. All chemicals were used as received without further purification.

Phosphate-buffered saline (PBS, 1x) (1 L) was prepared by mixing 8 g of NaCl, 1.44 g of Na_2_HPO_4_, 270 mg of KH_2_PO_4_, and 200 mg of KCl. BHI media was prepared according to the instructions on the chemical bottle. Luria Bertani (LB) (1 L) (pH 7.0) was prepared by mixing tryptone (10 g), yeast extract (5 g), and NaCl (10 g). Milli-Q water (resistivity = 18 MΩ·cm) was used to prepare all the media, buffer, and chemicals. All buffers and media were sterilized before use.

### 2.2. Methods

#### 2.2.1. Bacterial Growth Conditions

A microaerophilic *C. jejuni* NCTC 11168 was used as the target analyte, whereas *L. monocytogenes* Scott A and ser. Typhimurium-291RH were used as nontarget analytes. *C. jejuni* NTCT 11168 was grown on BHI agar at 37 °C under microaerobic conditions (85% N_2_, 10% CO_2_, 5% O_2_). A single colony of ser. Typhimurium-291RH and *L. monocytogenes* Scott A were inoculated in 3 mL of BHI liquid media separately and incubated at 37 °C overnight, at 200 rpm. A 500 µL aliquot of the overnight cultures of ser. Typhimurium-291RH and *L. monocytogenes* Scott A were inoculated into 50 mL of fresh liquid BHI medium separately and were incubated at 37 °C in an incubator shaker until they reached the mid-log phase. The spread plate technique was used to enumerate and express the bacteria in CFU/mL.

#### 2.2.2. Extraction, Purification, and Confirmation of the FlaGrab

CC-FlaGrab tagged with GST was expressed in *E. coli* BL21, induced expression with IPTG, and was purified using the fast protein performance chromatography (FPLC) system as described previously, except that proteins were eluted in 10 mM reduced L-glutathione in 1x; PBS [42]. A 12.5% sodium dodecyl sulfate polyacrylamide (SDS-PAGE) gel was used to confirm the size of the extracted protein.

#### 2.2.3. Measuring the Concentration of the Protein

The Pierce™ BCA Protein Assay Kit (Thermo Scientific, Waltham, MA, USA) was used to measure the concentration of the protein. Then, 25 µL of each standard or unknown sample was pipetted into a microplate well (working range = 20–2000 µg/mL). This was followed by adding 200 µL of the working reagent to each well and mixing the plate thoroughly on a plate shaker for 30 s. The plate was covered and incubated at 37 °C for 30 min. Finally, the plate was cooled down, and the absorbance was taken at 562 nm using a plate reader.

#### 2.2.4. Growth Clearance Assay

A bacterial growth clearance assay was carried out as previously described [29] with the following modifications: BHI broth was used in place of NZCYM. Also, 5 mL of (OD_600_ of 0.35) bacterial growth was used instead of 3 mL. The bacterial culture grew under microaerobic conditions until OD_600_ = 0.5. Then, 200 µL of the culture was used instead of 100 µL when mixed with 5 mL of sterile 0.6% molten BHI agar at 55 °C. Lastly, 10 µL of UV-sterilized CC-FlaGrab solution in 10 mM reduced L-glutathione/PBS was spotted onto the agar at the following concentrations: 2.92 mg/mL, 1.46 mg/mL, 0.292 mg/mL, and 0 mg/mL.

#### 2.2.5. Preparation of MWCNT Dispersion and Fabrication Protein Immobilized Electrode

The homogenous MWCNT dispersion was prepared by adding 1 mg of MWCNTs to 1 mL of DMF and ultrasonicated for an hour at 40 W using an ultrasonic homogenizer (Omni International, SONICRAPTOR 250, Kennesaw, GA, 30144 USA). A glassy carbon electrode (GCE) was polished using 0.05 μm alumina powder on a polishing pad for 5 min and then sonicated for 5 min in a bath sonicator to remove the adhered polishing powder. Later, the electrode was rinsed with deionized water (DI water), kept in the oven at 70 °C for 45 min until completely dry, and allowed to cool down at room temperature. Once it cooled down, 1.5 µL of the MWCNT dispersion was drop-cast on the GCE electrode surface and allowed to dry for 15 min at room temperature, and then it was dried at 70 °C for 5 min in the oven. Once the electrode dried, it was transferred to an ice bucket and cooled down before immobilizing the protein on the electrode. PBSE, a molecular tethering molecule, was used to immobilize the protein onto the MWCNT-modified electrode. For this process, the MWCNT-modified electrode was rinsed with DMF, then 4.5 µL of 10 mM PBSE was drop-cast on the electrode and incubated for 15 min. Then, excess PBSE was rinsed with DMF and followed by 1x PBS (pH 7.4). In the next step, 5 µL of 1 mg/mL CC-FlaGrab protein (in 1x PBS buffer) was drop-cast on the electrode surface and incubated for 24 h at 4 °C to immobilize the protein on the electrode surface. Later, it was rinsed with 1x PBS buffer to remove any unbound proteins. In the next step, 0.1% BSA solution in 1x PBS was dropped on the electrode surface and incubated for 30 min to block any unmodified electrode surfaces. Lastly, the electrode was thoroughly washed and incubated for 15 min with 1x PBS buffer.

#### 2.2.6. Electrochemical Impedance Measurements

The impedimetric characterization was conducted using the potentiostat (Model: CHI-920C) by CH Instruments Inc., located in Austin, TX, USA. The electrochemical system used in this experiment comprised three electrodes: a working electrode, a reference electrode, and a counter electrode. Electrochemical impedance spectroscopy (EIS) measurements were carried out using a 5 mM [Fe(CN)_6_]^4−^/Fe(CN)_6_]^3−^ redox couple, across a frequency range of 1 Hz to 10 kHz with an amplitude of 5 mV. The measurement results were presented as % R_CT_, where

(1) $$\%\;R_{CT}=\frac{R_{CT,measured}-R_{CT,baseline}}{R_{CT,baseline}}\times 100\%$$

#### 2.2.7. Sensitivity, Specificity, and Stability Tests

Different concentrations of *C. jejuni* (10^2^ CFU/mL to 10^8^ CFU/mL) were prepared and tested with an RBPP-immobilized biosensor for sensitivity measurement. Before impedimetric electrochemical impedance measurements, 50 µL of the *C. jejuni* solution was incubated with an RBPP-immobilized electrode for 12 min in a microaerophilic environment. Finally, the electrode was washed with 1x PBS buffer, and impedimetric measurements were taken. The specificity of the CC-FlaGrab protein was evaluated using 10^7^ CFU/mL of either the target or nontarget pathogen. The stability of the biosensor was evaluated after fabricating the electrode with CC-FlaGrab. RBPP-immobilized electrodes were stored at 4 °C in 1x PBS solution for one day, three days, one week, and two weeks, and were tested using 100 CFU/mL of *C. jejuni* after each period. R_CT_ was measured, and % R_CT_ was calculated from the experimental data with the response one day later. The measurement error was computed for every period that was tested in duplicate.

#### 2.2.8. Validation Tests Using a Chicken Cecal Sample

The chicken cecal sample was used as an ex vivo sample for the experiment. The cecal samples were obtained from the Southern Poultry Research Group, Georgia, and were surgically removed. In the next step, cecal contents were extracted aseptically and resuspended in equal-weight sterile PBS. In the initial step, 100 µL of the cecal sample was diluted with 900 µL of PBS buffer. Then, 10 µL of Halt TM protease inhibitor cocktail (100x) (ThermoFisher Scientific) was added to the diluted cecal sample, mixed, and incubated at 37 °C for 30 min [43]. After incubation, the contents were further diluted 100-fold [43,44,45,46,47], and the diluted cecal sample was used to prepare different concentrations of *C. jejuni* 11168 (10^2^, 10^3^, 10^7^, and 10^9^ CFU/mL). In the next step, the electrochemical experiment was carried out using the EIS technique. As a first step, the receptor-binding phage protein-modified electrode was incubated with 50 µL of the diluted cecal sample, incubated for 8 min, washed with PBS buffer, and EIS measurement was taken as described in the Section 2. This reading was considered the baseline reading. In the next step, the electrode was rewashed with PBS buffer, and 50 µL of different concentrations of *C. jejuni* 11168 were drop-cast and incubated for 8 min. Then, EIS measurements were taken and ΔR_CT_ was calculated, as shown in Equation (2).

(2) $$\Delta R_{CT}\;(ohm)=(R_{CT,measured}-R_{CT,baseline})$$

A known concentration (10^5^ CFU/mL) of *C. jejuni* 11168 was used as a target analyte, whereas the same concentrations of *L. monocytogenes* Scott A and ser. Typhimurium-291RH were used as nontarget analytes. All three cultures were prepared in a diluted cecal sample. Finally, the EIS measurements were taken for the three cultures described earlier in the Section 2, and ΔR_CT_ was calculated as shown in Equation (2).

## 3. Results and Discussion

### 3.1. Confirmation of the Size, Concentration, and Activity of the Protein

The SDS-PAGE gel image shows that the protein molecular weight is approximately 62 kDa, including a 26 kDa GST tag (Supplementary Materials, Figure S1a,b). The protein concentration was 2.92 mg/mL after purifying it with a 50 kDa molecular weight cut-off column. Further, the agar growth clearance assay showed that the phage protein did not allow the *C. jejuni* 11168 to multiply, confirming that the extracted protein is active (Supplementary Materials, Figure S1c).

### 3.2. Fabrication and Working Principle of the Biosensor

**Figure 1:**
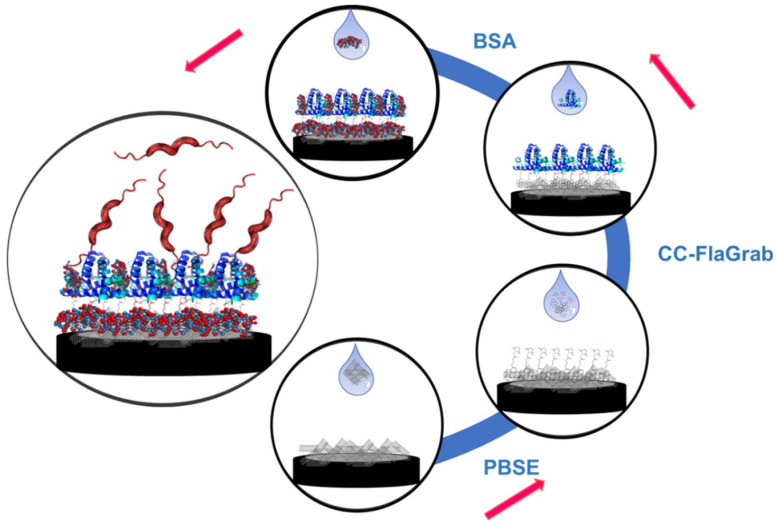
The electrode preparation process, including drop-casting the MWCNTs, cross-linker PBSE, phage protein CC-FlaGrab, and finally, adding 0.1% BSA as a blocking agent. Red color arrows represent the steps of the electrode preparation process.

A schematic representation shows the method used for immobilizing the phage protein for the detection of *C. jejuni* (Figure 1). The basal substrate, GCE, was modified by drop-casting with a thin layer of MWCNTs. MWCNTs have been widely used in transducers due to their unique structural and chemical properties in biosensing applications. MWCNTs are not only highly conductive, but also highly stable. In addition, the highly porous surface of MWCNTs provides a large electroactive area for binding various biological molecules, which increases biosensor sensitivity [48,49,50,51]. The high surface-to-volume ratio of MWCNTs allows for a high molecule charge per geometric unit, which helps signal amplification. In addition, it acts as the architecture for immobilizing the biorecognition element, phage protein. CC-FlaGrab needs to be immobilized strong enough to minimize the leaching of the protein while preserving its interaction with the bacterial receptor glycan molecule; any immobilization technique should not affect the critical amino acid involved in interacting with the bacterial receptor. The immobilization method should also consider the correct orientation and conformation of the protein for productive interaction. If the immobilization method could specifically target the GST tag for immobilization purposes instead of CC-FlaGrab, the adverse effects on the structure of the RBPP and critical amino acid would be minimized. The method should not only be compatible with the nanostructured probe to increase the surface area and sensitivity of the detection but should also be a method that does not require a complete understanding of the structural information of the protein.

PBSE has been successfully used in numerous biosensor applications with many types of proteins [22,24,25,51,52,53,54,55,56,57,58]. In this work, PBSE was used to immobilize the phage protein onto the MWCNT-modified electrode, covalently. PBSE acts as a cross-linker and attaches CC-FlaGrab through the linker to the surface of the MWCNTs. The aromatic pyrenyl moiety of the PBSE forms an irreversible π–π stacking and strongly attaches to the surface of the MWCNT. Since PBSE reacts with the N-terminal amine group, it allows us to orient the protein in a way that would allow productive interaction with the receptor. Since the GST tag is at the N terminus, the chemical reaction with the cross-linker would have minimal impact on the conformation or binding site of CC-FlaGrab. Since the protein is linked through a covalent bond to the linker, the chance of leaching out is minimized, and the protein would be stable at various pH and temperature ranges. In addition, CC-FlaGrab binds to acetamidino-modified pseudaminic acid, Pse5Ac7Am, displayed on the flagellin subunits of *C. jejuni*. Once the protein immobilization was complete, the *C. jejuni* was drop-cast on the electrode, and different concentrations of *C. jejuni* were incubated with the immobilized electrode. Finally, *C. jejuni* detection was accomplished by EIS by comparing the difference between R_CT_ before and after the target *C. jejuni,* and the phage protein was immobilized.

### 3.3. Electrochemical Characterization and Detection of the C. jejuni

#### 3.3.1. Sensitivity of the Biosensor

**Figure 2:**
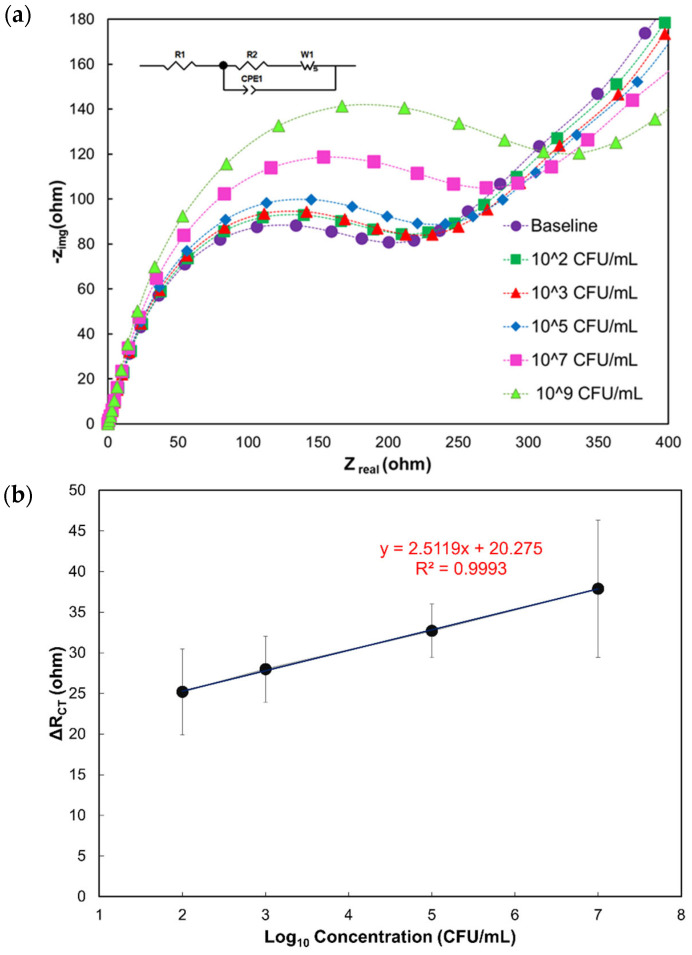
(**a**) Nyquist plots show the impedance response to varying concentrations of the target analyte *C. jejuni* 11168. The equivalent electrical circuit used for fitting the Nyquist data is shown in the inset. (**b**) Calibration curve showing a linear relationship between the differential charge transfer resistance ΔR_CT_ (ohm) and the logarithmic *C. jejuni* 11168 concentrations.

The impedimetric biosensing response of the phage-modified electrode was tested at different concentrations (10^2^, 10^3^, 10^5^, 10^7^, and 10^9^ CFU/mL) of *C. jejuni* in 1x PBS buffer. The magnitude of the semicircle (impedimetric response) increased with higher concentrations of *C. jejuni* (Figure 2a). Further, the data on charge transfer resistance (R_CT_) were used to create a calibration curve for analyte concentration (Figure 2b). The calibration curve indicates a nearly linear range of reliable detection between 10^2^ and 10^7^ CFU/mL. The limit of detection (LOD) was estimated using the Six Sigma method [59,60,61].

Experimental results reveal that the LOD of the phage protein-modified biosensor for *C. jejuni* is 10^3^ CFU/mL. This value is equivalent to the LOD of commercially available, highly sensitive, and expensive techniques (Table 2).

#### 3.3.2. Specificity of the Biosensor

**Figure 3:**
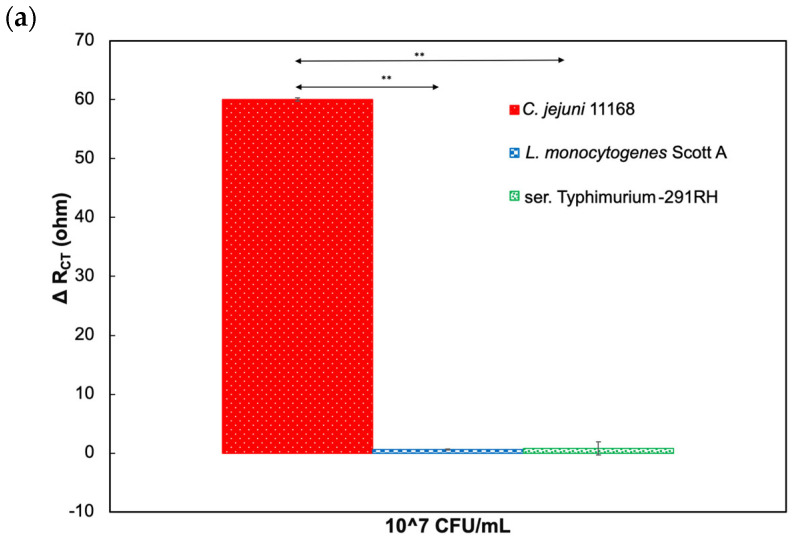

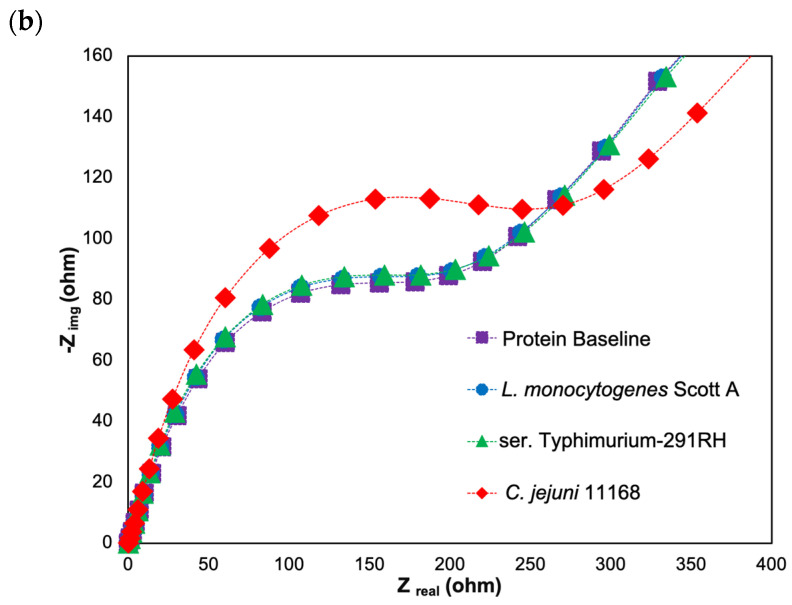
Response to target and nontarget pathogens in the presence of phage protein. (**a**) ΔR_CT_ (ohm) response values as the difference from baseline values with CC-FlaGrab protein. (**b**) Nyquist plot of the impedimetric response to target and nontarget bacterial cells in the presence of CC-FlaGrab protein. The asterisk indicates statistical significance (** p < 0.01, using One way-ANOVA with Post-hoc Tukey HSD test).

**Figure 4:**
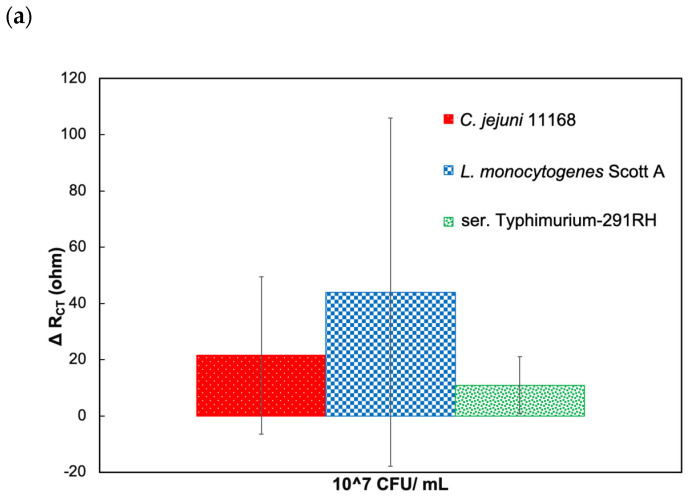

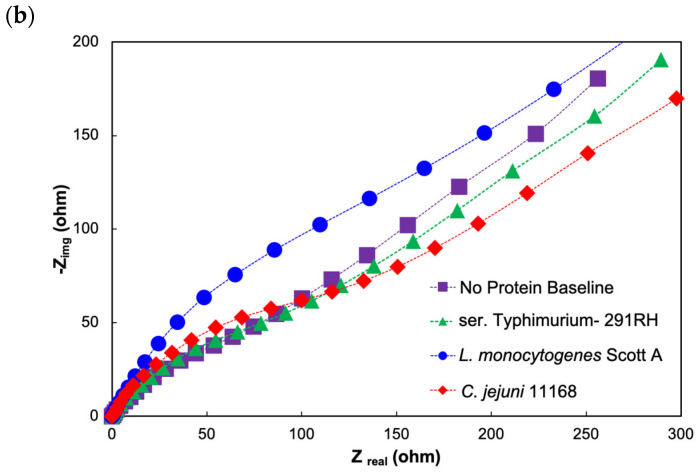
Response to target and nontarget pathogens in the absence of phage protein. (**a**) ΔR_CT_ (ohm) response values as the difference from baseline values when CC-FlaGrab is absent. (**b**) Nyquist plot of the impedimetric response to target and nontarget bacterial cells in the absence of CC-FlaGrab protein.

The Nyquist plots of target and nontarget bacterial cells in the presence and absence of CC-FlaGrab, respectively, demonstrate that the phage protein-modified electrode is nonresponsive to nontarget bacterial cells (*L. monocytogenes* Scott A and ser. Typhimurium-291RH) (Figure 3a,b). In contrast, a significant variation was observed for *C. jejuni* 11168. Further, in the absence of phage protein, the electrode showed very low R_CT_ change from the baseline for *C. jejuni* 11168 compared to nontarget pathogen *L. monocytogenes* Scott A (Figure 4a,b). Thus, the phage protein-based biosensor is specific only to target bacterial cells.

**Figure 5:**
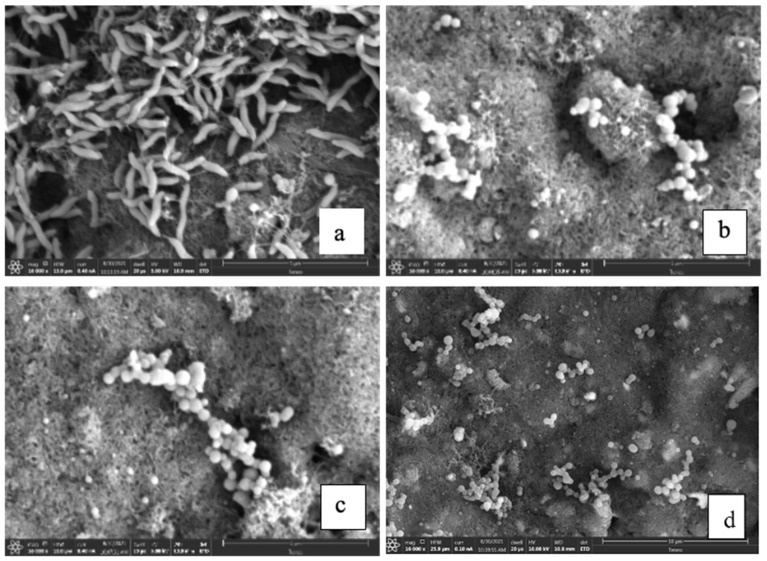
Phage protein CC-FlaGrab specificity in SEM with target and nontarget bacteria. Phage protein-modified electrode after exposure to *C. jejuni* 11168 (**a**), *L. monocytogenes* Scott A (**b**), ser. Typhimurium-291RH (**c**), and BSA molecule (**d**).

Further, SEM images confirmed that *C. jejuni* selectively attached to the fabricated biosensor architecture, whereas none of the *L. monocytogenes* 11168 and ser. Typhimurium-291RH cells (nontarget) attached to the biosensor architecture (Figure 5). These two data reveal that the developed phage protein (CC-FlaGrab)-based protein architecture is specific towards the target bacterial cell.

#### 3.3.3. Stability of the Biosensor

**Figure 6:**
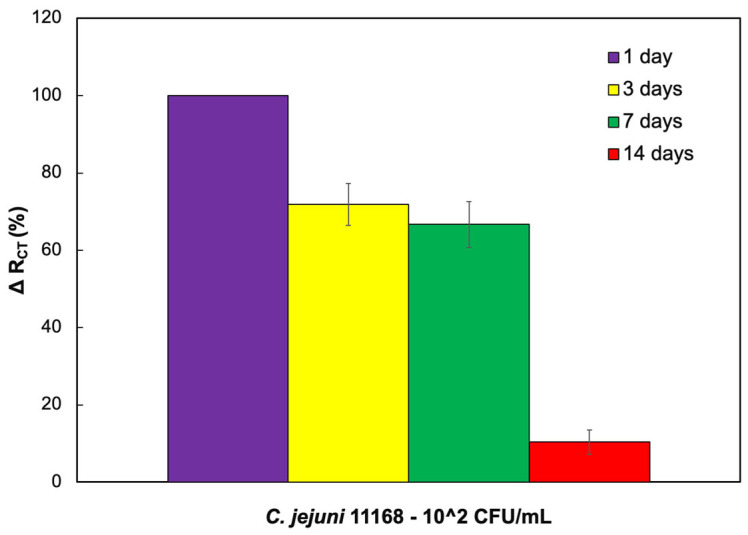
ΔR_CT_ (%) values represent the difference from the baseline value, which is measured within the first day of making the electrode.

After three days of preparation (Figure 6), the biosensor responses decreased by 29% with a 5% error margin. After a week, the signal decreased by 34% compared to the response obtained in one day, with a stable error of 5%. However, the sensor performance reduced to 10% after two weeks of electrode preparation, which clearly shows that the CC-FlaGrab protein’s activity degrades after one week and that the signals are minuscule.

### 3.4. Validating the Sensor with Chicken Cecal Samples

#### 3.4.1. Validating the Sensitivity of the Biosensor with Chicken Cecal Samples

The phage protein-immobilized electrode was tested with chicken cecal samples containing *C. jejuni* 11168 to evaluate the applicability of the phage protein-based biosensor. The ceca, a pair of blind-ended sacs that open off the large intestine, make up the largest portion of the chicken gastrointestinal tract (GIT). The cecum is the primary location for bacterial fermentation and pathogen colonization because of its larger and more diversified microbial population and longer digestion time (12–24 h). Studies in the literature report that the phyla Firmicutes, Bacteroidetes, and Proteobacteria are dominant in the chicken cecal microbiota [45,46,47,64,65,66,67,68,69,70,71,72]. Even though the cecal samples contain many bacterial cells, they do not contain *C. jejuni* 11168. In addition to the microbiota, cecal samples contain large amounts of proteases and metalloproteases [43,73,74]. Therefore, cecal samples were treated with a commercially available protease inhibitor cocktail before using the cecal samples in the detection experiments since these proteases have a high chance of degrading the phage proteins.

**Figure 7:**
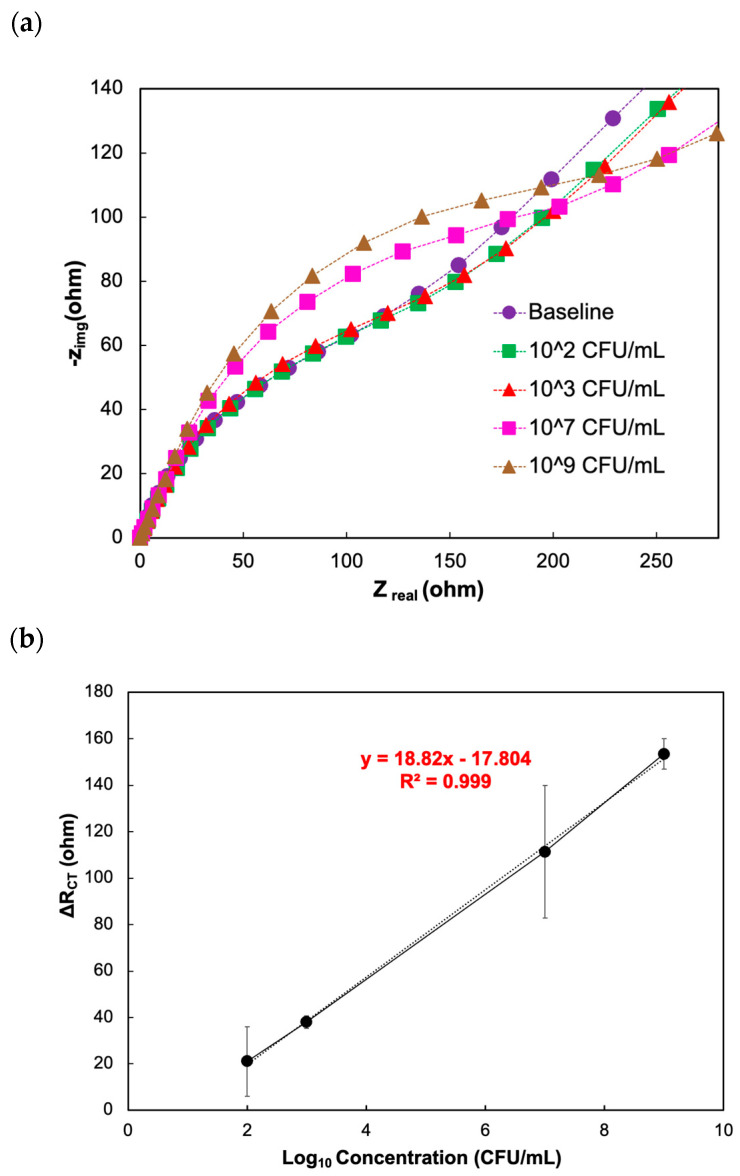
Response to the different concentrations of target pathogen in the chicken cecal sample in the presence of phage protein. (**a**) Nyquist plots show the impedance response to varying concentrations of the target analyte *C. jejuni* 11168 in the chicken cecal sample in the presence of phage protein. (**b**) Calibration curve showing a linear relationship between the differential charge transfer resistance ΔR_CT_ (ohm) and the logarithmic concentration of *C. jejuni* 11168 in the presence of phage protein on the electrode.

Further, cecal samples contain acetate, propionate, and butyrate produced through fermentation [70,75]. To minimize the background effect (nonspecific adsorption of chicken cecal components onto the electrode surface), the cecal samples were diluted 10^2^ times, and the following was performed: The diluted cecal sample was first incubated with the biosensor for 10 min, rinsed with PBS buffer, and then the EIS measurement was taken. This measurement was used as a baseline measurement [43,44,45,46,47,76]. In the next step, known concentrations of *C. jejuni* 11168 (10^2^ to 10^9^ CFU/mL) were spiked into the cecal samples and used for the sensitivity experiments. The R_CT_ values increased as the concentration of *C. jejuni* 11168 increased (Figure 7a). For this set of studies, the increment in R_CT_ could be defined as ΔR_CT_ = R_i_ − R_0_, where R_i_ is the R_CT_ value after incubation of *C. jejuni* 11168 cells, and R_0_ is the R_CT_ value after blocking with the diluted cecal samples that do not contain any target *C. jejuni* 11168 cells.

Additionally, a linear correlation exists between ΔR_CT_ and the bacterial concentration between 10^2^ and 10^9^ CFU/mL (Figure 7b). The LOD of *C. jejuni* 11168 is 10^2^ CFU/mL of the diluted cecal sample. This value is similar to the LOD values reported in the literature (Table 1; the LOD of our biosensor is equivalent to or better than the reported LOD of the commercially available highly sensitive techniques, including real-time PCR (Table 2)). These findings showed that the phage protein-immobilized electrode could detect *C. jejuni* 11168 cells in the cecal sample with high sensitivity. This is further supported in the literature describing other instances of biosensor detection of *C. jejuni* 11168. Compared to previously developed biosensors, our suggested biosensor offers a limit of detection and detection time that is equivalent to or supersedes those described previously (Table 1). Furthermore, additional optimizations, such as optimizing the number of washing steps, the concentration of the blocking agent, and the incubation time of the blocking agent, should be carried out to increase sensitivity and minimize errors.

#### 3.4.2. Validating the Specificity of the Biosensor with the Chicken Cecal Sample

**Figure 8:**
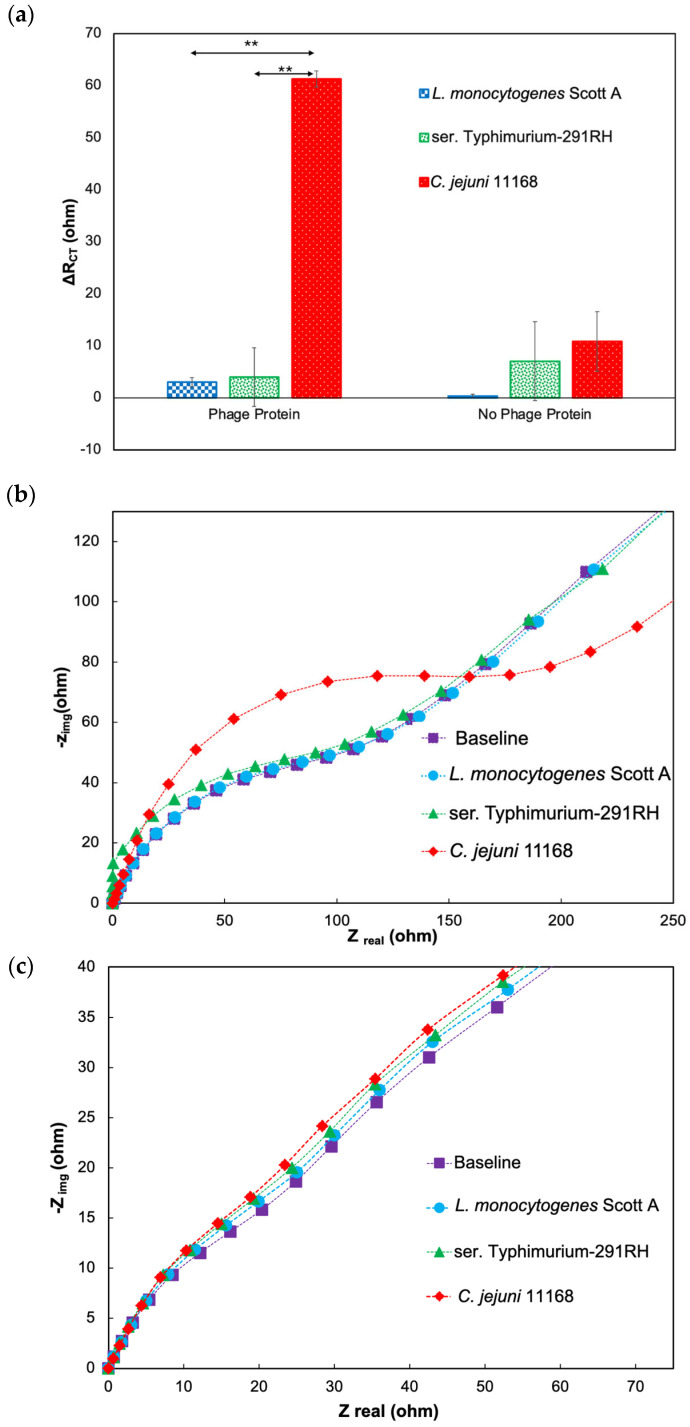
Response to target and nontarget pathogens in chicken cecal samples in the presence and absence of phage protein. (**a**) ΔR_CT_ (ohm) values of the response as the difference from baseline values with phage protein and no phage protein. (**b**) Nyquist plot of the impedimetric response to target and nontarget bacterial cells in the presence of phage protein. (**c**) Nyquist plot of the impedimetric response to target and nontarget bacterial cells in the absence of phage protein. The asterisk indicates statistical significance (** p < 0.01, using One way-ANOVA with Post-hoc Tukey HSD test).

*L. monocytogenes* Scott A and ser. Typhimurium-291RH were used as negative controls to evaluate the specificity of the developed biosensor. Known concentrations (10^5^ CFU/mL) of *C. jejuni* 11168, *L. monocytogenes* Scott A, and ser. Typhimurium-291RH cells were separately spiked into the diluted cecal samples and used for specificity studies. No obvious R_CT_ values were observed for either *L. monocytogenes* Scott A or ser. Typhimurium-291RH cells (Figure 8a). However, a large R_CT_ value was observed for *C. jejuni* 11168, demonstrating the high specificity of the phage protein-based biosensor and the eliminable physical adhesion of bacterial cells onto the electrode surface. According to the findings, the proposed biosensor offers great potential to be used as a sensitive and reliable *C. jejuni* 11168 detection method in food, clinical, and environmental diagnostics.

## 4. Conclusions

A highly specific novel phage protein-based biosensor was developed to detect *C. jejuni* in both in vitro and ex vivo samples. The sensor was developed by immobilizing a phage protein on a MWCNT-modified glassy carbon electrode using the cross-linker PBSE. The ex vivo samples were diluted with PBS buffer to minimize the background effect on the electrode matrix. The proposed biosensor exhibited a low detection limit of 10^2^ CFU/mL in ex vivo samples. The immobilized bioreceptor phage protein was highly specific and stable when it reacted with the target analyte in both in vitro and ex vivo samples. Further, the biosensor was highly specific towards *C. jejuni* NCTC 11168 as no signal was observed for ser. Typhimurium-291RH and *L. monocytogenes* Scott A. These features make it a promising tool for detecting bacterial cells in food, clinical, and environmental monitoring.

## Figures and Tables

**Table 1:** Established biosensors used for the detection of *C. jejuni*.

Detection Technique	Bioreceptor	Matrix	Analysis Time	Detection Range/Limit	References
Optical biosensor -SPR	Antibody	Apple juice	<1 h	1.1 × 10^5^ CFU/mL	[32]
Optical biosensor -SPR	Antibody	Milk	25 min	10^2^–10^9^ CFU/mL	[33]
Optical biosensor -SPR	Antibody	Washing water	NR	10^3^ CFU/mL	[34]
Optical biosensor -SPR	Antibody	Bacterial suspension	NR	4 × 10^4^ CFU/mL	[35]
Optical biosensor -SPR	DNA probe	Extracted DNA	NR	2.5 × 10^−9^ mol/L	[36]
Optical biosensor -SPR	DNA probe	Extracted DNA	NR	10^2^ copy/mL	[37]
Optical biosensor -SPR	Receptor-binding phage protein	Bacterial suspension	NR	10^2^ copy/mL	[31]
Colorimetric aptasensor	Aptamer	Chicken carcass	NR	7.2 × 10^5^ CFU/mL	[38]
Electrochemical -Amperometry	Antibody, phosphatase	Turkey carcass wash	2.5 h	10^2^–10^7^ CFU/mL LOD = 2 × 10^4^ CFU/mL	[39]
Electrochemical -Amperometry	Antibody	Milk	<1.5 h	1 × 10^3^–5 × 10^5^ CFU/mL LOD = 4 × 10^2^ CFU/mL	[40]
Electrochemical -Impedimetry	Antibody	Patient’s stool	NR	10^3^ CFU/mL	[41]
Electrochemical -Impedimetry	Phage protein (CC-FlaGrab)	Chicken cecal	10 min	10^2^ CFU/mL	This work

NR—Not reported.

**Table 2:** Characteristics of commercially available test kits to detect *C. jejuni*.

Method	Technique	Target Bacteria	LOD	References
DUPONT Qualicon Bax^®^ System Q7 Real-Time PCR	Real-Time PCR (Quantitative)	*C. jejuni* *C. coli* *C. lari*	10^4^ CFU/mL	[62]
ANSR^®^ for Campylobacter	Real-Time PCR (Qualitative)	*C. jejuni* *C. coli* *C. lari*	1 CFU/analytical unit	[63]

## Data Availability

The authors confirm that the data supporting the findings of this study are available within the article and its Supplementary Materials. The data that support the findings of this study are available from the corresponding author, Ramaraja Ramasamy, upon reasonable request.

## Supplementary Materials

The following supporting information can be downloaded at: https://www.mdpi.com/article/10.3390/bios14080402/s1. Figure S1: (a) SDS-PAGE after concentrating the purified CC-FlaGrab phage protein (b) Schematic diagram of the phage protein (CC-FlaGrab) (c) Agar plate growth clearance assay in the presence of undiluted CC-FlaGrab protein (CC-FlaGrab protein is labeled as Gp047 in the image) and diluted CC-FlaGrab protein in the buffer in two different ratios (1:10 and 1:1 ratio).
