# Supplementary material for: A Bacteriophage Protein-Based Impedimetric Electrochemical Biosensor for the Detection of Campylobacter jejuni
Source: Biosensors (Basel). 2024 Aug 21;14(8):402. doi: 10.3390/bios14080402 (PMC11352438; doi:10.3390/bios14080402)
Supplement: Supplementary file 1 [file biosensors-14-00402-s001.zip › biosensors-3112338-supplementary.pdf]

biosensors

MDPI

Supplementary Materials

A Bacteriophage Protein-Based Impedimetric Electrochemical Biosensor for the Detection of *Campylobacter jejuni*

Baviththira Suganthan [1], Ashley M. Rogers [2,3], Clay S. Crippen [2,3], Hamid Asadi [1], Or Zolti [1], Christine M. Szymanski [2,3] and Ramaraja P. Ramasamy [1,*]

[1] Nano Electrochemistry Laboratory, School of Chemical, Materials and Biomedical Engineering, University of Georgia, Athens, GA 30602, USA; baviththira.suganthan@uga.edu (B.S.); or.zolti@uga.edu (O.Z.)
[2] Department of Microbiology, University of Georgia, Athens, GA 30602, USA; ashley.rogers123@uga.edu (A.M.R.); cszymans@uga.edu (C.M.S.)

[3] Complex Carbohydrate Research Center, University of Georgia, Athens, GA 30602, USA
[*] Correspondence: rama@uga.edu

(a)

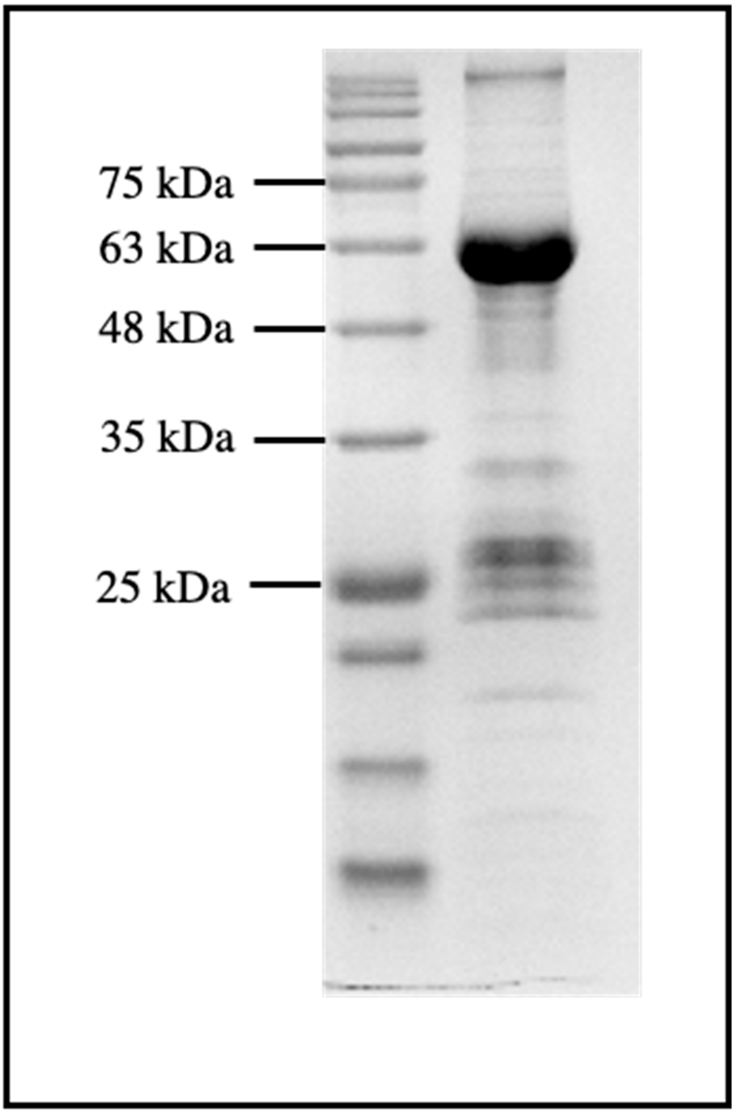

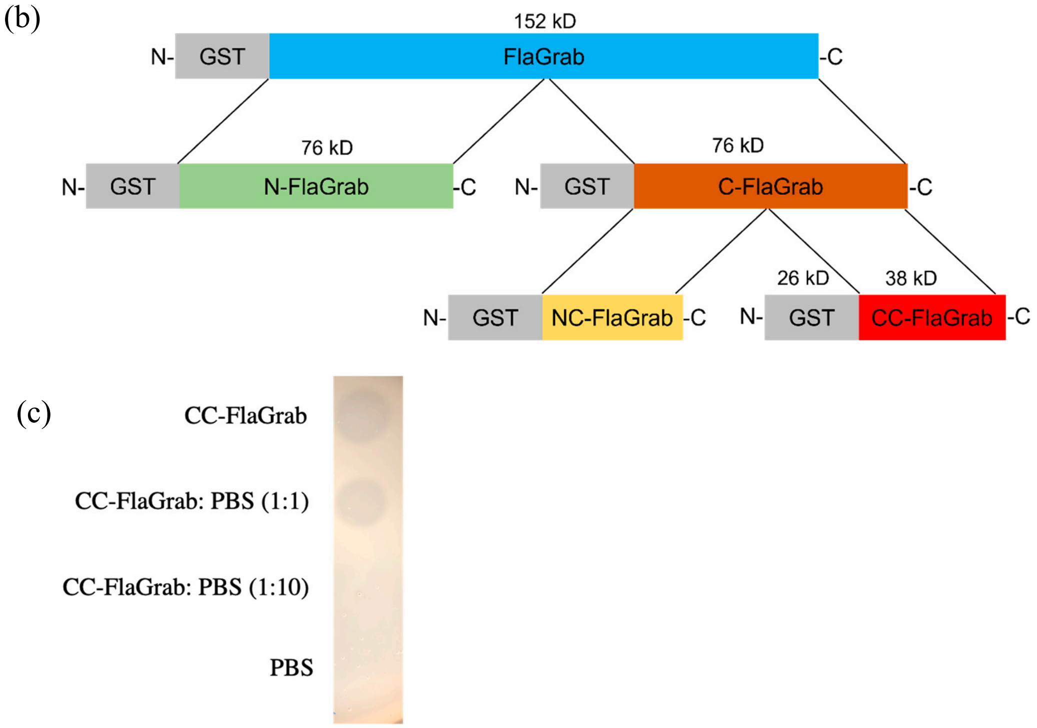

Figure S1. (a) SDS-PAGE after concentrating the purified CC-FlaGrab phage protein (b) Schematic diagram of the phage protein (CC-FlaGrab) (c) Agar plate growth clearance assay in the presence of undiluted CC-FlaGrab protein (CC-FlaGrab protein is labeled as Gp047 in the image) and diluted CC-FlaGrab protein in the buffer in two different ratios (1:10 and 1:1 ratio).
